# An exploratory qualitative study of patient mobility in the Lao People’s Democratic Republic

**DOI:** 10.1186/1472-6963-14-S2-P32

**Published:** 2014-07-07

**Authors:** Jo Durham, Visanou Hansana, Poom Viengnakhone, Vanphanom Sychareun

**Affiliations:** 1 School of Population Health, University of Queensland, Brisbane, Queensland, Australia; 2 University of Health Sciences, Faculty of Postgraduate Studies, Vientiane, People’s Democratic Republic of Lao

## Background

Most health system research by default conceptualises the health system as being contained within the geo-political borders of nation states. Yet health seeking strategies often span international borders. Based on research in the Lao People’s Democratic Republic (PDR), a lower-middle income country in South East Asia, this paper explores the circumstances in which patients decide to cross national borders for health care. It develops a typology of patient mobility in the context of a lower-middle income country undergoing rapid demographic and epidemiological transition. It provides concrete examples of patient agency, inequality and at least partial government support for patient movement.

## Materials and methods

This study used an exploratory, qualitative design. The sampling method followed a case series, purposive sampling design in that only people who had been overseas for health care were included. Qualitative interviews (N = 35) were undertaken using a semi-structured thematic interview guide, informed by the literature. While an interview guide was used, the questions were structured so as to allow participants the opportunity to focus on the issues that were important to them.

## Results

Patients crossed borders for preventive, curative and specialist care, management of chronic disease and sexual and reproductive health. Respondents in our study who sought health care overseas, made decisions based on a number of factors including costs, convenience, experiences of friends and family, perceived quality of care and diagnostic ability, ease of communication with practitioners, presence of social or familial networks who could facilitate access to doctors, and type of treatment sought. The ability to cross the border conferred valuable benefits to patients but on the other hand, the presence of the border determined who accessed this care and at what price.

## Conclusions

The patients in our study were embedded in more than one national health context. The phenomenon of patient mobility illustrates the limitations of conceptualising health systems as being contained within the nation state. How for example, can we understand health-seeking strategies if these strategies take place in a space which spans geo-political borders and in what ways does it exacerbate existing inequalities in both sending and receiving countries? Patient cross-border movement challenges researchers and policy-makers to take a more holistic view of health systems as inclusively defined by the World Health Organisation.

